# Drooling, Swallowing Difficulties and Health Related Quality of Life in Parkinson’s Disease Patients

**DOI:** 10.3390/ijerph18158138

**Published:** 2021-07-31

**Authors:** Gladis Yohana Arboleda-Montealegre, Roberto Cano-de-la-Cuerda, César Fernández-de-las-Peñas, Carlos Sanchez-Camarero, Ricardo Ortega-Santiago

**Affiliations:** 1 Escuela Internacional de Doctorado, Universidad Rey Juan Carlos, Alcorcón, 28922 Madrid, Spain; gladisl12@hotmail.com; 2 Department of Physical Therapy, Occupational Therapy, Rehabilitation and Physical Medicine, Universidad Rey Juan Carlos, Alcorcón, 28922 Madrid, Spain; cesar.fernandez@urjc.es (C.F.-d.-l.-P.); carlos.camarero@urjc.es (C.S.-C.); ricardo.ortega@urjc.es (R.O.-S.)

**Keywords:** deglutition, deglutition disorders, drooling, Parkinson disease, quality of life, sialorrhea

## Abstract

Background: Parkinson’s disease (PD) is the most common neurodegenerative disorder associated with motor and nonmotor symptoms. Drooling, one of the nonmotor symptoms, can be present in 70–80% of patients with PD. The aim of this paper is to study the characteristics of PD patients with drooling compared to those without in terms of age, gender, disease duration, stage of the disease, swallowing difficulties, and health-related quality of life; methods: a cross-sectional study was conducted. The sample was divided into two groups: PD with drooling (*n* = 32) and PD without drooling (*n* = 30). Age, gender, disease duration and Hoehn & Yahr (H & Y) stage, Sialorrhea Clinical Scale for Parkinson’s Disease (SCS-PD), the 10-item Eating Assessment Tool (EAT-10), and the 39-item Parkinson’s Disease Questionnaire (PDQ-39) were compared between groups; Results: 62 individuals with PD, 40 men and 22 women (mean age 73 ± 8 years), were included. Overall, 32 patients reported drooling, and 30 did not exhibit it. The ANCOVA found significant differences between groups for the EAT-10 score (0.83, 95% CI = 5.62–9.03; *p* = 0.016) and SCS-PD score (1.48, 95% CI = 0.86–6.81; *p* < 0.001). Analysis of the PDQ-39 scores revealed no significant differences between groups for the PDQ-39 total score (*p* > 0.057) and in all subscales. The inclusion of gender, age, disease duration, and H & Y as covariates did not influence the results (all *p* > 0.05). Conclusions: drooling is related to swallowing difficulties assessed with EAT-10 but not with health-related quality of life assessed with PDQ-39 in PD patients with drooling compared to PD patients without it. Age, gender, duration of the disease, and the H & Y state of PD patients with and without drooling seem to be similar.

## 1. Introduction

Parkinson’s disease (PD) is the second most common neurodegenerative disorder, with an estimated prevalence at 0.3% of the general population and being approximately 1% in those over 60 years of age worldwide [1,2]. This disorder affects 1–5% of individuals aged 65–69 years of age and 1–3% of those above 80 years of age. The estimated incidence is from 8 to 18 per 100.000 inhabitants/year worldwide. Patients with PD typically suffer from a wide range of nonmotor symptoms such as cognitive disorders, constipation, excessive sweating, fatigue, mood disorders, pain, or sexual problems [3]. These signs and symptoms impair the performance of their daily activities, reducing their level of independence [4].

Among these nonmotor problems, drooling is defined as excessive pooling and poor control of saliva in the oral cavity that might be caused by impaired salivary clearance [5] and is a frequent manifestation of PD. It can be present in 70–80% of PD patients, and its cause is linked to an impaired automatic ability to swallow, secondary to hypokinesia or due to a motor side effect of certain drugs, particularly the neuroleptics [6]. In addition, drooling presents a negative impact for both patients and caregivers.

Traditionally, drooling was related to age, disease duration, disease severity, and a worse quality of life of PD patients. The Sialorrhea Clinical Scale for Parkinson’s Disease (SCS-PD), which evaluates the frequency and intensity of drooling through seven items, as well as the functional and social impact, was the most accepted scale due to its validity and information about PD patients’ perspectives about drooling [6,7].

The aim of this paper was to compare the characteristics of PD patients with and without drooling in terms of swallowing difficulties and health related quality of life (HRQoL). Our initial hypothesis is that drooling could be related to swallowing difficulties assessed with EAT-10 but not with HRQoL in PD patients compared to those without drooling.

## 2. Materials and Methods

### 2.1. Study Design

A cross-sectional study was conducted at the Rey Juan Carlos University (Madrid, Spain) with outpatient PD subjects from Movement Disorder Units from Comunidad de Madrid (Spain). The Strengthening the Reporting of Observational Studies in Epidemiology (STROBE) Statement guidelines were followed.

### 2.2. Participants

Consecutive individuals with PD who fulfilled the modified diagnostic criteria of the Brain Bank of the United Kingdom [8] and were diagnosed by experienced neurologists were enrolled in the current study. The inclusion criteria were patients in stages I, II, III and IV of the Hoehn & Yahr (H & Y) scale; patients whose motor response to pharmacological treatment was stable; patients who presented complications of therapy, such as dystonia and dyskinesias, and those who were not receiving specific treatment for drooling at the time of the study.

The study exclusion criteria were the diagnosis of neurological diseases other than PD by medical records; the inability to understand instructions and actively cooperate in the tasks indicated, based on a score ≤24 in the Mini-Mental State Examination (MMSE) [9]; stage V of the H & Y scale, or a history of psychiatric problems and depression.

### 2.3. Procedure

The sample was divided into two groups: patients with drooling and patients without drooling, determined by the diagnosis of a neurological expert on movement disorders using the United Parkinson’s Disease Rating Scale (UPDRS) part II (item 6). Patients with 3 or more points were allocated into the group with drooling. Patients with 2 or less points were allocated into the group without drooling.

The study was conducted in accordance with the national and international ethics standards. Ethical approval was granted by the Local Ethics Committee (0406201910119). Informed consent was given in writing by all participants before inclusion in the study, and all procedures were conducted according to the Declaration of Helsinki.

### 2.4. Measures

A demographic and clinical questionnaire including age, gender, disease duration, and H & Y stage was registered for all patients by experienced neurologists.

The SCS-PD was used as the primary measure. The SCS-PD scale was originally written, administered, and validated in Spanish [10]. The SCS-PD score is a seven-item, retrospective, self-administered clinical scale, and it measures the relationship between drooling and meal times, degree of diurnal and nocturnal drooling, difficulties speaking or eating, rate of severe drooling, and degree of social discomfort. Values range from 0 (minimum) to 21 (maximum) intensity. SCS-PD was validated using saliva volume measurements in PD patients and healthy volunteers in prior research. In our study, patients were instructed to complete the survey based on symptoms present during the previous week [6,7,10]. Spouses or caregivers were invited to help patients, but it was stressed that the survey related mainly to patients’ thoughts and feelings (as illustrated in Supplementary Table S1).

The 10-item Eating Assessment Tool (EAT-10) [11] was used. This is a clinical questionnaire to document the initial dysphagia symptom severity in patients with swallowing disorders. It is rapidly administered, simply calculated, and has an easy-to-use scale that has excellent internal consistency, test-retest reliability, and criterion-based validity. The EAT-10 is also used in clinics worldwide, and the Spanish version was used in the present study. The EAT-10 includes 10 questions about the severity of symptoms of oropharyngeal dysphagia. Each question is scored from 0 to 4 (‘no problem’ to ‘severe problem’). The total T-EAT-10 score is calculated by adding up the scores of each question, and higher scores indicate the self-perception of a high level of dysphagia severity [12] (Supplementary Table S2).

HRQoL was assessed with the Spanish version of the 39-item Parkinson’s Disease Questionnaire (PDQ-39). The PDQ-39 was the first specific questionnaire for the evaluation of the HRQoL in PD patients and comprises 39 questions, each with 5 multiple-choice answers related to the frequency of the disease manifestation. The answers refer to the impact of the illness on the patient’s life in the previous month, as was explained to the patient before the interview. The 39 questions are divided into 8 dimensions: mobility (10 questions), activities of daily living (ADL) (6), emotional well-being (6), stigma (4), social support (3), cognition (4), communication (3), and bodily discomfort (3). The score for each question ranges from zero (0) to four (4): “never” = 0; “occasionally” = 1; “sometimes” = 2; “often” = 3; “always” = 4. Each dimension score ranges from 0 to 100 in a linear scale, in which 0 is the best and 100 the worst quality of life [13] (as illustrated in Supplementary Table S3). EAT-10 and PDQ-39 were analyzed as secondary measures.

All questionnaires were fulfilled within two hours of the administration of anti-Parkinson’s medication, during the ‘on’ phase of the medication cycle in the morning, as this is the period during which patients do most of their daily activities, by one examiner who had experience with how to carry out the tests and was blinded to the patient’s condition.

### 2.5. Sample Size Determination

The sample size determination and power calculations were performed with a G*power 3.1 (Franz Faul, Universitat Kiel, Kiel, Germany). The calculations were based on a Cohen d index of 1 [14], a 2-tailed test, an α level of 0.05, and a desired power of 95%. The estimated desired sample size was at least 27 individuals per group. This was increased by 10% in each group for possible data loss.

### 2.6. Statistical Analysis

Data were analyzed with the SPSS statistical package (21.0. version) (IBM Corporation, Armonk, NY, USA). Results are expressed as mean and 95% confidence interval (95% CI). The Kolmogorov–Smirnov test revealed a normal distribution of all variables (*p* > 0.05). Demographic characteristics of both groups were compared using an unpaired Student *t*-test and χ^2^ tests of independence.

A one-way ANCOVA with group (PD with drooling or PD without drooling) as the between-subjects factor, and gender, age, disease duration, and H & Y as covariates, was used to investigate the differences in drooling symptoms, swallowing disorders and quality of life. The Pearson correlation test (r) was used to determine the association between SCS-PD and the clinical variables. The statistical analysis was conducted at a 95% confidence level, and a *p* value < 0.05 was considered statistically significant. The standardized mean difference (SMD) was calculated by dividing the between-group difference by the pooled standard deviation to enable comparison of effect sizes. Values were considered as trivial when ranging from 0.0 to 0.2, small from 0.2 to 0.49, moderate from 0.5 to 0.79, and large when >0.8.

## 3. Results

### 3.1. Demographic and Clinical Data of the Sample

A total of 62 individuals with PD, 40 men and 22 women (mean age 73 ± 8 years), were included. The mean duration of the disease of the total sample was 9.0 ± 6.4 years (95% CI, 7.3–10.6 years) and the mean H & Y stage was 2.3 ± 0.8 (95% CI, 2.1–2.5). A total of 32 patients (51.6%) reported drooling symptoms (item 6 of UPDRS score ≥3), and 30 (48.4%) did not exhibit drooling. No differences in the age (*t* = −0.017, *p* = 0.987), gender (χ^2^ = 1.371, *p* = 242), duration of the disease (*t* = 0.856, *p* = 0.396), and H & Y state (*t* = 1.493, *p* = 0.141) existed between groups. Table 1 summarizes the demographic and clinical data of the groups.

### 3.2. Primary Measure

The one-way ANCOVA revealed significant differences between groups for SCS-PD (group: F = 71.983, *p* < 0.001). Patients with drooling exhibited higher scores than patients without drooling in terms of SCS-PD. The inclusion of gender, age, disease duration, and H & Y as covariates did not influence the results (all *p* > 0.05) (as illustrated in Table 1).

### 3.3. Secondary Measures

The ANCOVA found significant differences between groups for the EAT-10 score (group: F = 6.148, *p* = 0.016). However, the inclusion of gender, age, disease duration, and H & Y as covariates did not influence the results (all *p* > 0.05). Table 1 summarizes EAT-10 scores for both study groups.

Analysis of the PDQ-39 scores revealed no significant differences between groups in PDQ-39 total score (F = 3.771, *p* = 0.057) and in all subscales (Mobility: F = 0.265, *p* = 0.608; ADL: F = 0.414, *p* = 0.522; emotional well-being: F = 0.066, *p* = 0.797; stigma: F = 1.275, *p* = 0.263; social support: F = 0.645, *p* = 0.425; communication: F = 2.329, *p* = 0.132; cognition: F = 1.818, *p* = 0.183; bodily discomfort: F = 3.973, *p* = 0.051): patients with drooling exhibited similar scores than patients without drooling. The inclusion of gender, age, disease duration, and H & Y as covariates did not influence the results (all *p* > 0.05). Table 1 summarizes PDQ-39 scores for both study groups.

### 3.4. SCS-PD and Clinical Features in PD Patients

No significant correlations between clinical variables and SCS-PD were found (all *p* > 0.145) in both groups. Table 2 summarizes the associations between SCS-PD and clinical features in patients with PD with and without drooling.

### 3.5. Intermeasure Comparisons of Effect Size

Large effects were observed between patients with and without drooling comparisons for SCS-PD (SMD = 1.48, 95% CI = 0.86–6.81) and EAT-10 (0.83, 95% CI = 5.62–9.03).

## 4. Discussion

Our results show that drooling is related to swallowing difficulties assessed with EAT-10 but not with HRQoL in PD patients compared to those without drooling. The inclusion of age, gender, duration of the disease, and the H & Y state of PD patients with and without drooling seem to be similar.

Factors typically associated with drooling are aging, severity of PD, duration of PD, the sum of the scores of UPDRS part II and III greater than 28 points, dysarthria, dysphagia, orthostatic hypotension, and a history of using antidepressants [15]. However, to the best of our knowledge, few studies investigated drooling in PD patients compared to that of a sample of PD patients without drooling. Our results show that drooling is related to swallowing difficulties but not to HRQoL. Traditionally, drooling was linked to a negative impact for both patients and caregivers, and PD patients with drooling were related to a poor quality of life [15,16]. Nevertheless, in our study HQRoL is not related to the presence of drooling in PD patients.

Healthy subjects produce approximately 1000 to 1500 mL of saliva each day. It was thought that sialorrhoea is caused by hypersecretion of saliva, but patients with PD produce less saliva per day than healthy subjects (approximately 750 mL each day) [17]. Thus, drooling was typically associated with infrequent swallowing and poor facial and oral neuromuscular control. Our results show that drooling is related to swallowing disorders assessed with EAT-10 in PD patients compared to PD patients without drooling. Further, other factors not assessed in our research could compound the problem of drooling and swallowing disorders, such as flexed head posture, open mouth posture, difficulties closing the lips, dental caries and gingivitis, medication, neuromuscular dysfunction, sensory dysfunction, edentate state or anatomic abnormalities as macroglossia, and facial or oropharyngeal weakness [17,18]. Even though PD patients with drooling had higher EAT-10 scores than those without drooling, the EAT-10 scores were overall fairly low across both groups. This could be linked to our inclusion criteria of PD patients between stages I–IV in the H & Y, with a mean severity stage of II observed for both groups.

Although drooling seems to be related to a swallowing dysfunction in PD patients [19], other authors reported that drooling is present in 86% of PD subjects with dysphagia, but only 44% of subjects without dysphagia [20], supporting the theory that impaired swallowing contributes to drooling, but is not the unique cause in PD. Furthermore, some authors show that PD patients who do not appear to have dysphagia clinically, may still have impaired swallowing [18]. In our study, swallowing difficulties were assessed with the EAT-10. However, it would be interesting if future studies could contrast these findings after using objective measurement methods, such as the modified barium swallow (MBS) testing or videofluoroscopy, comparing PD patients with and without drooling, since symptom scores from the EAT-10 might not correspond to swallowing pathophysiology [21].

Drooling was previously described as a troubling problem for PD patients, often giving rise to social embarrassment [22,23]. Surprisingly, our results show that PD patients with drooling do not present a worse HRQoL. In this respect, Leibner et al. [24] compared the HRQoL of PD droolers and nondroolers with age-matched controls. Among PD droolers, only 18.8% stated that accumulation of saliva did not bother them in social situations; 18.8% claimed that they knew other people may notice the saliva but that they could control it; 3.1% said that they stopped attending social meetings altogether. Of those PD nondroolers, there were none that had any of the above problems with social situations due to saliva accumulation. As in our study, there was no significant difference in the overall quality of life between droolers and nondroolers, and consequently, our results are in line with Leibner et al. [24], as no relationship was found between PD patients with and without drooling in terms of HRQoL. The whole sample recruited in our study presented an average H & Y of 2.3 score (2.4 score in PD with drooling and 2.1 score in PD without drooling). Usually, PD patients at stage 2 are affected by a moderate and compensated form of PD, so drooling and swallowing problems are mainly variables, and the clinical evaluation is often normal. The lack of difference between quality-of-life assessment could be due to the moderate severity of the drooling for both groups.

Previous reviews [15] identified possible factors associated with drooling, such as severity of PD, male gender, aging, hallucinations, duration of the disease, dysarthria, orthostatic hypotension, and a history of using antidepressants. Our results show that gender, age, duration of the disease, and H & Y state of PD patients with and without drooling are similar. Our results are in line with Leibner et al. [24] in terms of age, disease duration, and disease severity, and with Kalf et al. [25], who demonstrated that patients with drooling difficulties presented a mean duration of disease of seven years with no difference in disease duration between droolers and nondroolers. A possible explanation for this finding is the ambiguity of the duration of disease term identified in prior studies [24,25]. Also, a possible difference in medication between groups may be behind the lack of differences in the present study.

Our study presents several limitations. Firstly, our PD patients meet inclusion criteria and, therefore, extrapolation of our results to other PD conditions or populations should be conducted with caution. Secondly, it would be interesting to incorporate in future studies an objective measure to assess swallowing difficulties and to determinate the baseline swallowing frequency rate, as swallowing difficulties were determined using a subjective scale. In addition, all assessments were carried out in the “on” period, so no extrapolations should be made to the “off” period, as drooling is more prominent during this period. Besides, future studies should include the UPDRS score in “off” and “on” to detect or not possible correlations with SCS-PD, EAT-10, PDQ-39 or some subscores of the UPDRS scale, such as hypomimia and voice that could be present in patients with drooling. Thirdly, future studies should include information about Levodopa Equivalent and the eventual dosage of Levodopa Equivalent and Dopamine Agonists or other PD medications to study a possible correlation with drooling and swallowing. Finally, the rate of saliva production is known to vary by time of day, so this also needs to be taken into consideration when comparing our results with future studies.

## 5. Conclusions

Our results show that drooling is associated with swallowing difficulties as assessed with EAT-10 and that drooling does not affect the quality of life as assessed with PDQ-39 in people with PD, compared to those without drooling. Age, gender, duration of the disease, and the H & Y state of PD patients with and without drooling seem to be similar. Future studies should consider our findings to proportionate integral rehabilitation programs to people with PD with drooling disorders.

## Figures and Tables

**Table 1:** Clinical and demographic features of patients with Parkinson’s disease with and without drooling.

	PD with Drooling (*n* = 32)	PD without Drooling (*n* = 30)
Gender (M/F)	23/9	18/12
Age (years)	73 ± 8 (69–77)	73 ± 7 (69–75)
Disease duration (years)	9.6 ± 7.2 (7.3–12.4)	8.7 ± 5.5 (5.9–10.1)
Hoehn and Yahr stage	2.4 ± 0.8 (2.1–2.7)	2.1 ± 0.8 (1.7–2.4)
SCS-PD *	8.4 ± 4.3 (6.8–9.9)	1.0 ± 1.8 (0.3–1.7)
EAT-10 *	5.4 ± 7.8 (2.6–8.2)	1.6 ± 3.1 (0.4–2.7)
PDQ-39 (0–100)	29.8 ± 12.6 (25.4–34.3)	24.3 ± 9.3 (20.7–27.8)
Mobility	41.2 ± 25.4 (32.2–50.2)	37.8 ± 25.9 (27.9–47.7)
ADL	33.9 ± 22.1 (26.1–41.8)	30.5 ± 20.5 (22.6–38.3)
Emotional well-being	33.0 ± 17.2 (26.9–39.2)	31.8 ± 18.9 (24.6–39.1)
Stigma	14.2 ± 7.2 (7.1–21.0)	9.7 ± 4.1 (5.6–13.8)
Social support	6.8 ± 4.2 (2.1–11.4)	4.6 ± 3.3 (1.7–7.6)
Communication	30.5 ± 22.1 (22.7–38.4)	22.4 ± 19.5 (14.9–29.1)
Cognition	40.3 ± 18.1 (33.9–46.7)	31.0 ± 17.1 (24.5–37.5)
Bodily Discomfort	38.6 ± 22.3 (30.7–46.5)	28.2 ± 18.7 (28.4–35.1)

Data are expressed as means ± standard deviation (95% confidence interval); M: male; F: female; EAT-10: 10-item Eating Assessment Tool; SCS-PD: Sialorrhea Clinical Scale for Parkinson’s disease; PDQ-39: 39-items Parkinson’s disease Questionnaire; ADL: activities of daily living. * Significant differences between PD patients with and without drooling (*p* < 0.001).

**Table 2:** Associations between Sialorrhea Clinical Scale for Parkinson’s disease and clinical variables in patients with and without drooling.

	Patients with PD with Drooling	Patients with PD without Drooling
Age (years)	r = 0.229; *p* = 0.199	r = 0.184; *p* = 0.330
Disease duration (years)	r = −0.076; *p* = 0.675	r = 0.152; *p* = 0.653
Hoehn and Yahr stage	r = −0.001; *p* = 0.997	r = 0.264; *p* = 0.145
EAT-10	r = −0.032; *p* = 0.861	r = 0.092; *p* = 0.629
PDQ-39 total score	r = 0.037; *p* = 0.836	r = −0.091; *p* = 0.609
Mobility	r = −0.032; *p* = 0.861	r = −0.033; *p* = 0.861
ADL	r = 0.058; *p* = 0.747	r = 0.066; *p* = 0.727
Emotional well-being	r = 0.088; *p* = 0.626	r = −0.052; *p* = 0.784
Stigma	r = −0.206; *p* = 0.249	r = 0.231; *p* = 0.220
Social support	r = −0.027; *p* = 0.883	r = 0.021; *p* = 0.911
Communication	r = 0.211; *p* = 0.239	r = 0.120; *p* = 0.281
Cognition	r = 0.150; *p* = 0.405	r = 0.249; *p* = 0.184
Bodily Discomfort	r = −0.061; *p* = 0.737	r = 0.084; *p* = 0.658

PDQ-39: 39-items Parkinson’s disease Questionnaire; ADL: activities of daily living.

## Data Availability

The authors confirm that the data supporting the findings of this study are available within the article and its supplementary materials.

## Supplementary Materials

The following are available online at https://www.mdpi.com/article/10.3390/ijerph18158138/s1. Table S1. Sialorrhea Clinical Scale for Parkinson’s Disease (SCS-PD). Spanish version, Table S2. Eating Assessment Tool (EAT-10). Spanish version, Table S3. Parkinson’s Disease Questionnaire (PDQ-39). Spanish version.
