# Supplementary material for: Drooling, Swallowing Difficulties and Health Related Quality of Life in Parkinson’s Disease Patients
Source: Int J Environ Res Public Health. 2021 Jul 31;18(15):8138. doi: 10.3390/ijerph18158138 (PMC8345955; doi:10.3390/ijerph18158138)
Supplement: Supplementary file 1 [file ijerph-18-08138-s001.zip › ijerph-1310543-supplementary.pdf]

<u>Sialorrhea Clinical Scale for PD (SCS-PD)</u>

Score is total of scores for each item (A-G).

Please read the following questions and try to answer them on the basis of how you felt during the past week.

A. During the day, when do you feel there is more saliva in your mouth?

0 = Never.
1 = At meal times.
2 = Throughout the day, not related to meals.
3 = All the time, even when I am asleep.

B. When you are asleep, how much saliva is there in your mouth?

0 = I don’t notice an increase in saliva.
1 = I notice increased amounts of saliva in my mouth, but my pillow doesn’t get wet.
2 = My pillow gets wet.
3 = My pillow and other bedclothes get wet.

C. While you are awake,

0 = I don’t drool.
1 = Saliva wets my lips.
2 = Saliva accumulates on my lips, but I don’t drool.
3 = I drool.

D. Does accumulation of saliva in your mouth impair your speech?

0 = No.
1 = I must swallow frequently to avoid difficulties.
2 = I have trouble speaking.
3 = I can’t speak at all.

E. Does accumulation of saliva in your mouth impair your eating ability?

0 = No.
1 = I must swallow frequently to avoid difficulties.
2 = I have trouble eating.
3 = I can’t eat at all.

F. How many times do you drool during the daytime?

0 = Never.
1 = Not more than 3 times.
2 = Often. I have to carry a handkerchief with me all the time.
3 = Permanently.

G. When you go out or on social occasions, does saliva accumulation bother you?

0 = No.
1 = I notice an accumulation, but it does not bother me.
2 = I realize other people notice it, but I can control the situation (for example, with a handkerchief).
3 = I have stopped attending social meetings.

Eating Assessment Tool (EAT-10)

How to complete this Questionnaire:

- This questionnaire helps to measure swallowing difficulties.
- These are statements many people have used to describe difficulty swallowing / eating
- To what extent do you experience the following problems?
- Circle the most appropriate response for each statement.

0 - 4 Rating Scale 0 = No problem 1 = Mild Problem 2 = Mild to moderate 3 = Moderate problem 4 = Severe problem

My swallowing problem has caused me to lose weight. 0 1 2 3 4
My swallowing problems interferes with my ability to go out for meals. 0 1 2 3 4
Swallowing liquids takes extra effort 0 1 2 3 4
Swallowing solids takes extra effort. 0 1 2 3 4
Swallowing pills takes extra effort. 0 1 2 3 4
Swallowing is painful 0 1 2 3 4
The pleasure of eating is affected by my swallowing. 0 1 2 3 4
When I swallow food sticks in my throat. 0 1 2 3 4
I cough when I eat. 0 1 2 3 4
Swallowing is stressful 0 1 2 3 4

TOTAL 10 x 4 = 40 max _______________

Parkinson’s Disease Quality of Life Questionnaire (PDQ-39)

Due to having Parkinson’s disease, how often during the last month have you.... *Never, Occasionally, Sometimes, Often, Always*:

1 Had difficulty doing the leisure activities which you would like to do?
2 Had difficulty looking after your home, e.g. DIY, housework, cooking?
3 Had difficulty carrying bags of shopping?
4 Had problems walking half a mile?
5 Had problems walking 100 yards?
6 Had problems getting around the house as easily as you would like?
7 Had difficulty getting around in public?
8 Needed someone else to accompany you when you went out?
9 Felt frightened or worried about falling over in public?
10 Been confined to the house more than you would like?
11 Had difficulty washing yourself?
12 Had difficulty dressing yourself?
13 Had problems doing up your shoe laces?
14 Had problems writing clearly?
15 Had difficulty cutting up your food?
16 Had difficulty holding a drink without spilling it?
17 Felt depressed?
18 Felt isolated and lonely?
19 Felt weepy or tearful?
20 Felt angry or bitter?
21 Felt anxious?
22 Felt worried about your future?
23 Felt you had to conceal your Parkinson's from people?
24 Avoided situations which involve eating or drinking in public?
25 Felt embarrassed in public due to having Parkinson's disease?
26 Felt worried by other people's reaction to you?
27 Had problems with your close personal relationships?
28 Lacked support in the ways you need from your spouse or partner?
29 Lacked support in the ways you need from your family or close friends?
30 Unexpectedly fallen asleep during the day?
31 Had problems with your concentration, e.g. when reading or watching TV?
32 Felt your memory was bad?
33 Had distressing dreams or hallucinations?
34 Had difficulty with your speech?
35 Felt unable to communicate with people properly?
36 Felt ignored by people?

37 Had painful muscle cramps or spasms?
38 Had aches and pains in your joints or body?
39 Felt unpleasantly hot or cold?
